# Studying the social dimension of emotion regulation

**DOI:** 10.3389/fpsyg.2013.00922

**Published:** 2013-12-05

**Authors:** Vera Shuman

**Affiliations:** Department of Economics, University of LausanneLausanne, Switzerland

**Keywords:** emotion regulation, social context, emotion, anticipated emotions, social emotions, social cognition, mimicry

Emotion regulation has traditionally been studied as an individual phenomenon. Increasingly, however, researchers are developing the theoretical concepts and empirical paradigms needed to study emotions and their regulation in social contexts.

Emotion regulation is a *social* phenomenon in multiple ways: (1) Social contexts stir and steer emotions, (2) a common objective of emotion regulation is to change social situations, (3) the communication of emotions is a means to regulate emotions, and (4) emotion regulation and social cognition are linked.

Researchers who empirically study emotion regulation in social contexts use a variety of paradigms and methods, including self-report, EEG, fMRI, psychophysiological measures, voice parameters, facial action coding, and economic games. Subject populations are community samples, students, adolescents, employees, and patients. Emotions in interactions with strangers, co-workers, friends, and family members are examined.

The research topic “The social dimensions of emotion regulation” showcases the breadth of approaches to studying emotions in social contexts, discerns common themes, and proposes avenues for future research. I first provide a brief overview of the 16 articles featured in the research topic and then describe how the articles bridge traditional and future directions of emotion regulation research.

## Overview

### Social contexts stir and steer emotions

Social contexts regulate emotions by arousing emotions and by determining the goals of emotion regulation. In their review of the cultural regulation of emotions, De Leersnyder et al. (2013) provide examples for how different social contexts give rise to distinct emotions: at the individual, relational, and structural level, cultures afford particular emotional situations and appraisal tendencies that lead to different emotions. von Scheve (2012) complements this review with a theory paper that connects psychological concepts from emotion regulation research with sociological concepts from research on emotion work. He points out that an individual's material, cultural, and social resources within a society influence the occurrence of emotional situations; additionally, framing rules guide the interpretation of a situation, which can then lead to an emotion.

Going beyond a direct “social context arouses emotion” relation, two experimental studies in this research topic demonstrate that the behavior of others interacts with an individual's current emotion regulation approach to produce emotions. Specifically, Grecucci et al. (2012) show in an economic game study how fair and unfair offers from others influence individuals' emotions differently when individuals engage in the emotion regulation strategies of mentalizing or distancing. The EEG study by Mella et al. (2012) suggests that the emotional reactions to others' pain depend on emotion regulation capacities that change between adolescence and adulthood.

Additionally, social contexts regulate emotions by determining the goals of emotion regulation. As reviewed by De Leersnyder et al. (2013) and by von Scheve (2012), this is evident in different emotion regulation goals across cultures and by different feeling rules for groups within cultures. Empirically, Snyder et al. (2013) find an association of the regulation goals in participants at a particular cultural event, “Burning Man,” and the likelihood of using different emotion regulation strategies to achieve the normative emotional state in the cultural context.

### A common objective of emotion regulation is to change social situations

Emotion regulation is also social when the goals of the emotion regulation are to influence interaction partners. As a result of the emotion regulation, individuals create or modify social situations.

Three survey studies find that individuals regulate their emotions to influence others' feelings and behavior. Simons et al. (2012) show that crying is down-regulated for interpersonal reasons. Wong et al. (2013) find that individuals who amplify their positive emotions at work are more likely to attain their goals in interactions with superiors but not with colleagues, whereas authentic expressions of positive emotions are associated with goal attainment in interactions with colleagues and superiors. Niven et al. (2012) demonstrate that higher variability in the regulation strategies that individuals use to influence others' emotions (“spin”) is negatively associated with affective well-being and relationship closeness.

Additionally, emotion regulation leads to the creation of social situations when individuals choose to behave toward others in specific ways because they seek to experience or not experience anticipated emotions. Two experimental studies examine individuals' anticipated emotions and decision making. In an economic game study, van der Schalk et al. (2012) find, for example that individuals who anticipate pride (regret) after making fair (unfair) offers to others decide to subsequently share resources with others more generously. Wagner et al. (2012) adapt a lottery paradigm to differentially arouse the interpersonal emotion guilt or the intrapersonal emotion regret. They then demonstrate effects of trait and state guilt on loss aversive behavior.

### The communication of emotions is a means to regulate emotions

Emotion regulation is social when emotions are communicated to others and thereby changed. The communication can be accomplished by verbal, vocal, and facial expressions of emotions. To what extent communicating emotions to others improves emotions depends on a number of factors, including how one shares the emotional event and how others react to the shared information.

In their study using self-report, voice parameters, and skin conductance, Matejka et al. (2013) find that individuals who face unpleasant stimuli believe that talking about facts is a more efficient emotion regulation strategy than talking about emotions. However, although talking about facts reduces negative valence during the emotional experience, talking about emotions reduces emotional arousal. A similar dissociation between the effects of emotional sharing on valence and arousal is observed by Seehausen et al. (2012) who study the influence of listeners' reactions on voice and psychophysiological parameters. A listener who paraphrases shared emotions induces less negative affect but more arousal in the sharer.

Facial mimicry is another way emotions are shared and regulated in social interactions. Fischer et al. (2012) observe individuals' natural tendency to mimic others in a social interaction with strangers and intimates. In support of a social contextual perspective, they demonstrate that mimicry is reserved for affiliative expressions (smiles) rather than negative expressions (disgust) when interacting with friends or family members; strangers' expressions were not mimicked in this study. How do individuals control whether or not they mimic somebody? In an fMRI study, Vrticka et al. (2013) identify the brain activity associated with expressing and suppressing mimicry when viewing dynamic facial expressions.

### Links between emotion regulation and social cognition

The social nature of emotion regulation is also evident in studies examining the link between social cognition abilities and emotion regulation tendencies. Rowland et al. (2012) demonstrate that in healthy adults, good social cognition is associated with the decreased use of maladaptive emotion regulation strategies. Schizophrenia and borderline personality disorder patients, however, differ from healthy controls in that they have lower mean levels of social cognition abilities and a higher likelihood for maladaptive emotion regulation strategies, and also in that they do not show the linkage between these variables, which is observed in healthy controls.

## Bridging traditional and future directions

All but three articles in the research topic cite the conceptual emotion regulation framework by James Gross and colleagues, demonstrating the high influence of this model on subsequent research (e.g., Gross, 1998). This model distinguishes several emotion regulation processes that the present papers address: situation selection and modification (van der Schalk et al., 2012; Wagner et al., 2012; De Leersnyder et al., 2013), attention deployment (Mella et al., 2012), cognitive change (appraisal/re-appraisal; Grecucci et al., 2012; De Leersnyder et al., 2013; Snyder et al., 2013), and response modulation (emotion expression/suppression/amplification; Fischer et al., 2012; Simons et al., 2012; Matejka et al., 2013; Snyder et al., 2013; Vrticka et al., 2013; Wong et al., 2013). In the empirical studies, an emotional event is traditionally viewed as separate from the regulation of the emotion in order to examine changes in the emotion.

However, authors who theoretically examine the dependence of emotions on the cultural context or the group context question the common approach to treat emotion generation and regulation as separate processes (De Leersnyder et al., 2013; Kappas, 2013). A constructive proposition that may bring the views closer together can be seen in von Scheve's contribution, which extends the Gross model by adding sociological concepts. As a result of this exercise, the complex influences of a larger social context on emotions and their regulation are taken into account. Future work will test the value of this extension of the classical model.

## Conclusion

The research topic brings together an exciting body of empirical research, reviews, and theoretical discussions. The papers demonstrate the significance of the social dimension of emotion regulation in many areas including social influence, conflict behavior, pro-social behavior, economic decision making, and mental health. Future research is needed to further validate and extend this work. In addition to increasing our knowledge on the four central themes outlined above, future research should tackle other topics concerning the social nature of emotion regulation, such as group emotions and emotions on the web (Kappas, 2013). The theoretical work (e.g., von Scheve, 2012) and new empirical paradigms (e.g., Wagner et al., 2012) presented in this research topic constitute valuable tools to guide future research on the social dimension of emotion regulation.
